# Dataset from spirometer and sEMG wireless sensor for diaphragmatic respiratory activity monitoring

**DOI:** 10.1016/j.dib.2019.104217

**Published:** 2019-07-05

**Authors:** Giorgio Biagetti, Virgilio Paolo Carnielli, Paolo Crippa, Laura Falaschetti, Valentina Scacchia, Lorenzo Scalise, Claudio Turchetti

**Affiliations:** a Department of Information Engineering, Polytechnic University of Marche, Ancona, Italy; b Maternal and Child Health Institute, Polytechnic University of Marche and Salesi Hospital, Ancona, Italy; c Department of Industrial Engineering and Mathematical Sciences, Polytechnic University of Marche, Ancona, Italy

**Keywords:** Diaphragm surface electromyographic signal, Spirometer signal, sEMG wireless sensor, Respiratory activity monitoring

## Abstract

We introduce a dataset to provide insights into the relationship between the diaphragm surface electromyographic (sEMG) signal and the respiratory air flow. The data presented had been originally collected for a research project jointly developed by the Department of Information Engineering and the Department of Industrial Enginering and Mathematical Sciences, Polytechnic University of Marche, Ancona, Italy. This article describes data recorded from 8 subjects, and includes 8 air flow and 8 surface electromyographic (sEMG) signals for diaphragmatic respiratory activity monitoring, measured with a sampling frequency of 2 kHz.

**Table:** Specifications table

Subject area	*Electrical and Electronic Engineering* *Biomedical Engineering*
More specific subject area	*Surface electromyography (sEMG)*
Type of data	*Data matrix, table, figure*
How data was acquired	*Air Flow signal: AD Instruments spirometer ML311 with MLT1000L respiratory flow head. sEMG signal: WiSEMG surface electromyography system with a wireless sensor.*
Data format	*Raw mat files*
Experimental factors	*Participants were familiarised with the experimental protocol by testing the equipment and software prior to recording.*
Experimental features	*Participants performed deep/normal breaths for an acquisition session of about 10 minutes. Air flow signal and Surface Electromyography signals were concurrently recorded during the voluntary activity.*
Data source location	*Ancona, Italy*
Data accessibility	*Data is within this article*
Related research article	*None*

**Table:** 

**Value of the data** • The data provide insights into the relationship between the diaphragm surface electromyographic (sEMG) signal and the respiratory air flow. • The findings might be on the focus of early detection scenario. • The data is suitable for different pattern recognition tasks such as respiratory activity variations or apnea detection. • The dataset can be used to investigate the capability to discover the activity of a deep muscle such as the diaphragm from sEMG signals.

## 1 Data

The dataset provided with this article supplies valuable information to investigate a correlation between the surface electromyographic signal (sEMG) acquired from the diaphragm muscle through the skin surface and the spirometer signal. The advantage of these data is to give a possibility to investigate the respiratory activity variations, or apnea detection, both from the electromyographic signal and the respiratory air flow [1], [2], [3], [4], [5].

The dataset consists in an archive file named “diaphragmatic_sEMG.zip”, containing 8 raw mat files, “S[1–8].mat”, corresponding to each recording session of each subject. The mat file contains three data matrices:

- “air_breathing”: contains the measure of the spirometer acquisition (time [s] and values [L/s]);
- “emg_breathing”: contains the measure of the sEMG signal acquisition (time [s] and values [mV]);
- “emg_resting”: contains the measure of the sEMG signal in rest condition for a possible calibration of processing algorithms or manipulation of the signals (time [s] and values [mV]).

The dataset contains recording sessions for a total duration of 3022 s, with a mean duration for each session of 377.75 s. Table 1 shows the details about the consistency of the dataset, in terms of duration.

**Table 1:** Data consistency: Acquisition time for each subject (breathing and resting activity).

Subject ID	Breathing activity [s]	Resting activity [s]
1	384	234
2	384	69
3	290	253
4	389	143
5	415	290
6	392	281
7	384	304
8	384	289

**Fig. 1:**
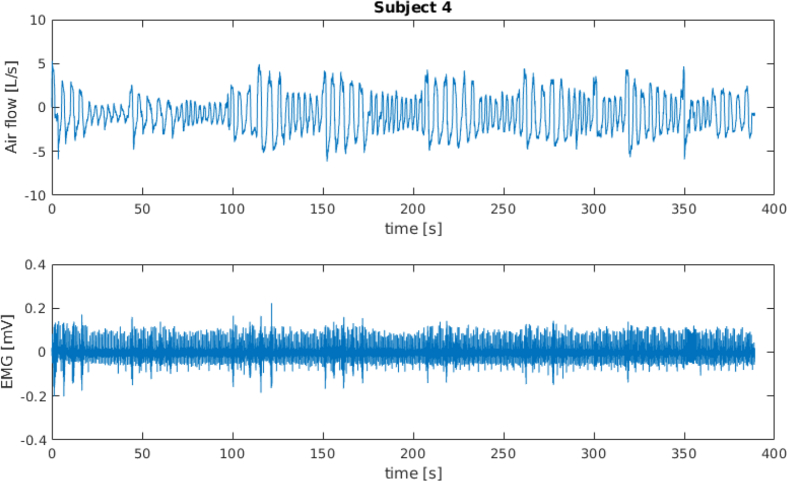
Data recorded from subject 4.

**Fig. 2:**
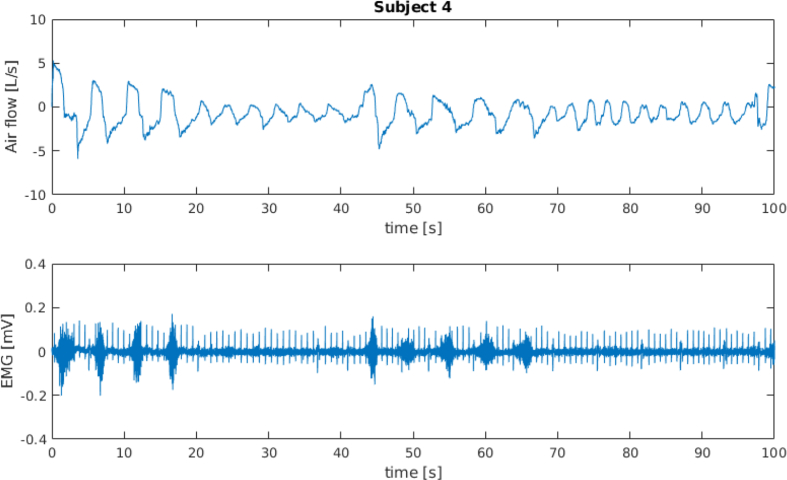
Data recorded from subject 4; first 100 s.

**Fig. 3:**
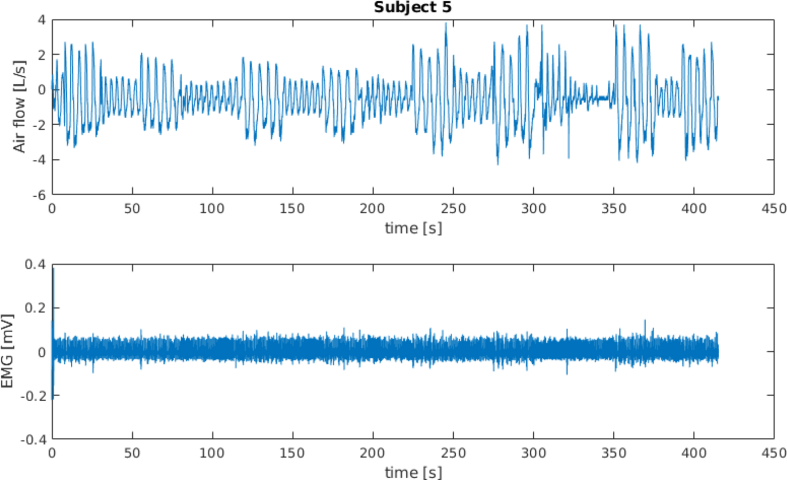
Data recorded from subject 5.

**Fig. 4:**
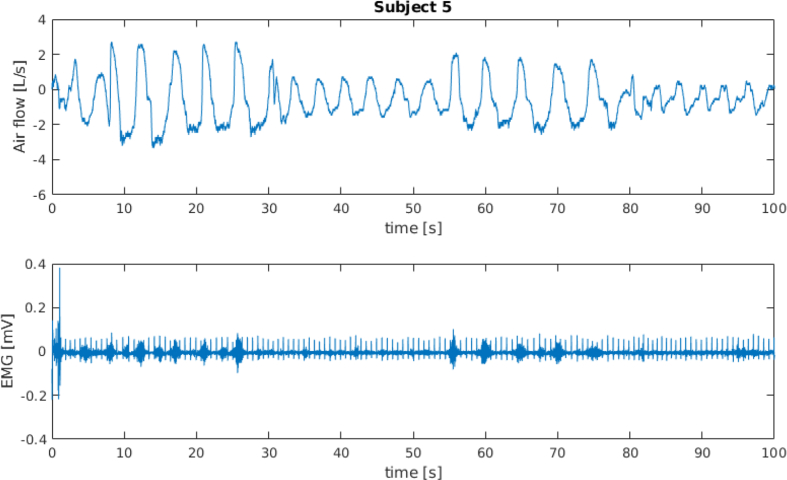
Data recorded from subject 5; first 100 s.

Fig. 1 and Fig. 3 show the spirometer and the sEMG signals for subject 4 and subject 5 (full time window and first 100 s), respectively. Fig. 2 and Fig. 4 show a frame of 100 s for the spirometer and the sEMG signal, respectively, of the same two subjects, where it can be seen how these signals are affected by electrocardiography (ECG) contamination. The “emg_resting” signals have been included in this dataset specifically to aid algorithms for the removal of this contamination.

## 2 Experimental design, materials, and methods

### 2.1 Partecipants

A total of 8 subjects that includes 5 males and 3 females aged between 23 and 30 years were recruited for participation as reported in Table 2.

- Age = 26.25 ± 3.5 years old
- BMI = 21.95 ± 1.9 kg/m^2^.

**Table 2:** Partecipants.

ID	Sex	Age	BMI [kg/m^2^]
1	female	24	18.5
2	female	24	22.0
3	male	26	25.0
4	male	33	21.9
5	male	24	21.7
6	male	23	22.2
7	female	26	20.8
8	male	30	23.5

The subjects were selected from healthy people (student volunteers).

A detailed written consent was obtained from all participants.

### 2.2 Procedure

Air flow signal and sEMG signals were concurrently recorded during the voluntary activity. For the air flow signal, a spirometer with data recording PowerLab 4/25T (AD Instruments) was used. For the sEMG signal, a surface electromyography acquisition system named WiSEMG [6] with a wireless sensor node was used. WiSEMG is a low-cost wireless system capable of acquiring both the sEMG and the ECG signals, using wearable sensors. Both signals were acquired with a sampling frequency of 2 kHz. It comprises a series of base stations and several wireless sensing nodes. The nodes transmit wirelessly the bio-signals to the base stations through a custom protocol based on the IEEE 802.15.4 standard. The base stations can be up to four and are connected via USB to a control PC, where data are stored and analyzed by a dedicated graphical user interface, though in this experiment only one base station was needed.

The data recording session is started by manually pressing a record button on both the instruments. The subject waited 2 s before producing a “starter” signal. The starter signal corresponds to a cough, which can be easily identified in both recordings and used to synchronize the data streams. After the starter, the subject performed 5 deep breaths (5 s between each one), then 5 normal breaths (5 s between each one) and then again 5 deep breaths (5 s between each one). After this session, the subject was asked to breathe normally for 30 s and then repeat the session. Lastly, the subject was asked to stay still and breathe normally without the spirometer for about 5 minutes (to record a baseline sEMG signal possibly useful for algorithm calibration and successive data processing).

**Fig. 5:**
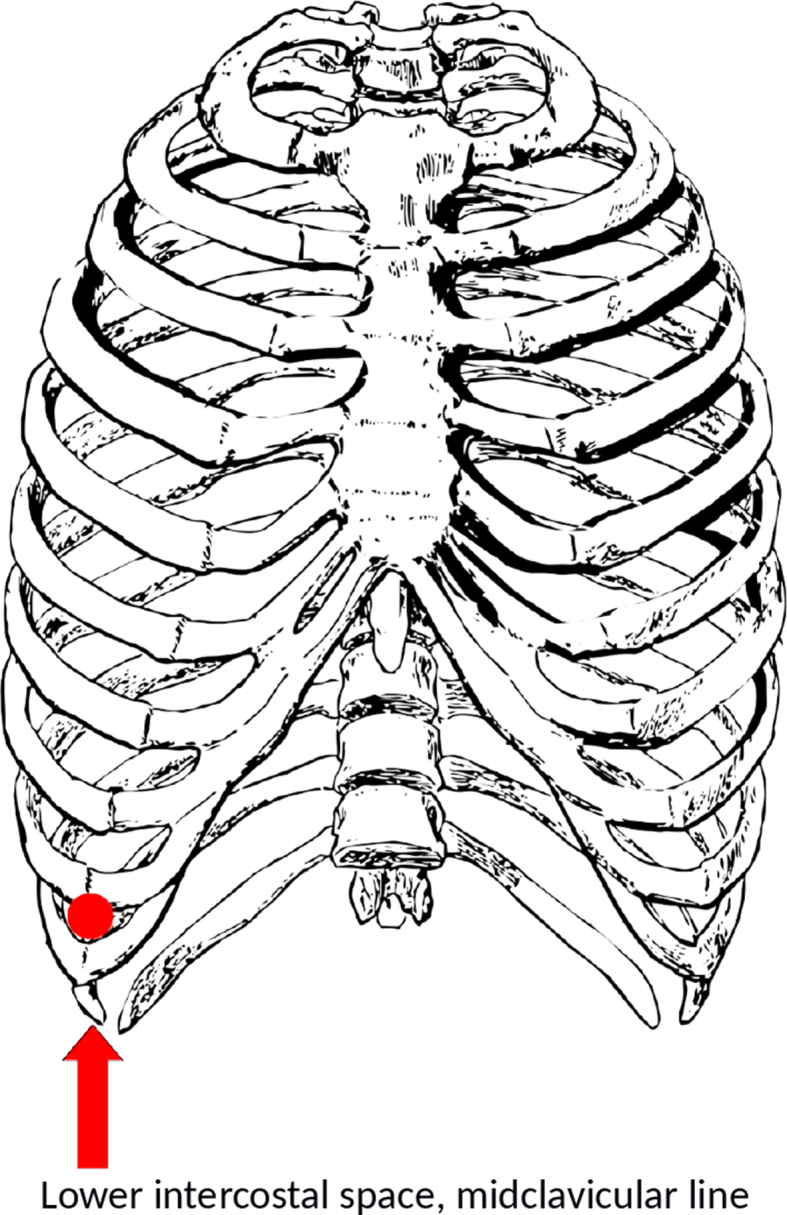
Node placement - anatomical reference (Pixabay Licence: https://pixabay.com/it/service/terms/#license).

In each session, the sensor node used to acquire the sEMG signal was placed in the lowest intercostal space, right side of the body, midclavicular line of the subject, as shown in Fig. 5. The WiSEMG system comprises a programmable gain amplifier (PGA), so the gain of each node was set to obtain the best signal specifically for each subject. The subject assumes the following positions: standing (during the breathing part), seated (during the last 5 minutes).
